# Maternal infections during pregnancy and offspring cognitive outcome: A nationwide full-sibling cohort study

**DOI:** 10.1371/journal.pmed.1004657

**Published:** 2025-06-24

**Authors:** Anders Husby, Kim D. Jakobsen, Jan Wohlfahrt, Mads Melbye

**Affiliations:** 1 Department of Epidemiology and Biostatistics, School of Public Health, Faculty of Medicine, Imperial College London, London, United Kingdom; 2 Department of Epidemiology Research, Statens Serum Institut, Copenhagen, Denmark; 3 Department of Congenital Disorders, Statens Serum Institut, Copenhagen, Denmark; 4 Department of Paediatrics and Adolescent Medicine, Rigshospitalet, Copenhagen, Denmark; 5 Department of Clinical Medicine, Aalborg University, Aalborg, Denmark; 6 Danish Cancer Institute, Cancer Epidemiology and Surveillance, Copenhagen, Denmark; 7 Danish Cancer Institute, Copenhagen, Denmark; 8 K.G. Jebsen Center for Genetic Epidemiology, Norwegian University of Science and Technology, Trondheim, Norway; 9 Department of Pediatrics, Stanford University School of Medicine, Stanford, California, United States of America; London School of Hygiene and Tropical Medicine, UNITED KINGDOM OF GREAT BRITAIN AND NORTHERN IRELAND

## Abstract

**Background:**

Maternal infections are common during pregnancy, but it is unclear how they impact the cognitive outcome of the offspring, with many studies suggesting adverse effects. Using long-term follow-up of a nationwide sibling cohort in Denmark with information on maternal antimicrobial prescriptions in community pharmacies and in-patient hospitalizations for infection, we aimed to estimate the effect of maternal infections during pregnancy on offspring school grades and intelligence test results in adolescence.

**Methods and findings:**

From population-based national registries we defined a cohort of all full-siblings, born from January 1, 1996 to December 31, 2,003 in Denmark, and linked them to maternal filled prescription for antimicrobial pharmaceuticals and maternal hospitalizations for infection during pregnancy. Standardized examination grades in language and mathematics at the final year of compulsory schooling, in addition to intelligence test scores (calculated as IQ) for a nested sub-cohort of full brothers, were used as outcomes. Among 274,166 children in the full-sibling cohort, 80,817 (29.5%) had a mother who during her pregnancy filled a prescription for a systemic antimicrobial, while 5,628 (2.1%) had a mother who during her pregnancy was hospitalized due to an infection. We found no consistent difference in school grades in language (z-score difference, 0.0, 95% confidence interval [CI] [−0.0,0.0]; *p* = 0.920) and mathematics (z-score difference, −0.0, 95% CI [−0.0,−0.0]; *p* = 0.042), and in IQ (IQ-difference, 0.3, 95% CI [−0.2,0.7]; *p* = 0.217), in children whose mother filled one antimicrobial prescription compared with children whose mother did not fill any, when taking shared family factors into account, while many associations were consistently significant when not taking shared family factors into account. Furthermore, we found no indication of an impact of maternal in-patient hospitalizations for infections during pregnancy on school grades (z-score difference for language, −0.0, 95% CI [−0.1,0.0]; *p* = 0.103; z-score difference for mathematics, 0.0, 95% CI [−0.0,0.0]; *p* = 0.809) or IQ (IQ-difference, 0.4, 95% CI [−0.8,1.6]; *p* = 0.545), when also taking shared family factors into account. Similar findings were found when considering infections in bi-weekly exposure periods during gestation. The main limitations of the study were lacking information on within hospital pharmaceutical prescriptions and the underlying pathogenic microorganisms.

**Conclusions:**

Our study does not support major effects of common maternal infections during pregnancy on offspring cognitive outcomes, and support the safety of commonly prescribed antimicrobials during pregnancy with respect to the long-term cognitive outcomes of the offspring.

## Author summary

**Why was this study done?:**

- Common maternal infections during pregnancy have been associated with offspring cognitive outcomes, but previous findings are conflicting.
- Only few of the previous studies investigating the association between maternal infections during pregnancy and cognitive outcomes used a sibling design to account for unmeasured familial confounding.
- Furthermore, previous studies investigated the influence of infections only in very broad exposure periods (i.e., during the whole pregnancy or during a trimester), potentially missing strong effects during short and critical developmental windows during gestation.

**What did the researchers do and find?:**

- Using a nationwide full-sibling cohort of children born in Denmark between 1996 and 2003, we investigated the association between maternal infections during pregnancy (identified by antimicrobial prescriptions or hospitalization for infection) and subsequent school grades in language and mathematics.
- For a nested full-brother cohort we additionally investigated the association between maternal infection during pregnancy and offspring IQ using results from an intelligence test conducted during evaluation for mandatory military service.
- The study found no consistent association between common maternal infections during pregnancy and offspring school grades or intelligence.

**What do these findings mean?:**

- The findings support a minimal impact of common maternal infections during pregnancy on cognitive abilities of the offspring.
- The findings indicate that there does not exist critical time windows for fetal brain development that are sensitive to common maternal infections during pregnancy.
- Future studies on the association between maternal infections and cognitive outcomes will benefit from using a sibling design to diminish unmeasured familial confounding.
- The main limitations of the study were lacking information on antimicrobials used during hospitalization and the underlying microorganisms responsible for the maternal infections.

## Abstract 3

Anders Husby and colleagues investigate the effect of maternal infections during pregnancy on offspring school grades and intelligence test results in adolescence.

## Introduction

It is well-known that specific rare infections during pregnancy can cause severe fetal brain damage and lead to life-long mental impairment [1–3]. However, it is unclear if common maternal infections, and the accompanying inflammatory response (e.g., fever), during short critical periods of fetal brain development, also restrict long-term cognitive development [4–16]. Using gestational week-specific information on all filled antimicrobial prescriptions during the fetal life of over 250,000 full-siblings, linked to nationwide information on school grades and intelligence tests in adolescence, we examined the influence of maternal infections on long-term cognitive outcome of the offspring.

Previous studies have mostly indicated a negative impact of maternal infections during pregnancy on offspring cognitive outcomes [4,10,12,14,15]. Specifically, negative effects of maternal infections on offspring cognitive outcomes have mainly been attributed to systemic infections and infections late in gestation [4,10,12]. However, a recent study analyzing the effect of maternal infections using a sibling design have suggested diminished negative effects compared to cohort studies using only covariate adjustments [13]. Additionally, the previous studies have been confined to investigating the effect of maternal infections in very broad exposure periods (i.e., the whole pregnancy or trimesters covering three months), potentially missing important short developmental windows during gestation. We hypothesize that specific short vulnerable periods for exposure to common infections during pregnancy could exist and that with highly detailed description of the specific exposure time period during prenatal life (i.e., the day of maternal antimicrobial prescription or the day of maternal in-patient hospital admission for an infection during pregnancy) we will be able to pinpoint such short periods.

Furthermore, we hypothesize that applying a full-sibling design, we will be able to take potential confounding, including unmeasured confounding by shared family factors, into account. Additionally, using prospectively collected information on all filled prescriptions and in-patient hospitalizations during pregnancy will remove recall bias and allow precise assessment of which gestational week of pregnancy the mother had an infection requiring treatment with systemic antimicrobials or hospitalization. Finally, taking advantage of registered intelligence test scores from evaluation for national military service for a nested sub-cohort of full-brothers, we will be able to estimate association between maternal infections during pregnancy and offspring IQ in a large population-based cohort, which is only rarely possible. These investigations could thus highlight if there is a potential for avoiding negative cognitive impacts of maternal infections during pregnancy on offspring outcome, which is important for both developed and developing countries.

In summary, we hypothesize that common maternal infections during pregnancy in short developmental windows could influence offspring cognitive outcomes. We investigate this hypothesis using a nationwide population-based full-sibling cohort with comprehensive prospectively collected data to avoid recall bias, low statistical precision, and unmeasured familial confounding.

## Methods

### Materials

The Danish Civil Registration System (CRS) provides every permanent Danish resident with a mandatory unique identification number, which is used for identification in nearly all areas of public life (e.g., health services, educational services, and military conscription) [17]. Furthermore, the CRS provides linkage between parents and children from the date of birth, allowing identification of siblings.

The Danish National Prescription Registry contains complete information on filled prescriptions in Denmark dispensed at all community pharmacies, starting from January 1, 1995. Every prescription is administered to a unique individual using the CRS identification number and the registry includes information on date of dispensing, Anatomical Therapeutic Chemical (ATC) code of the pharmaceutical, and form of dispensation (e.g., tablet or patch) [18]. Linkage of the prescription data to the Medical Birth Registry, which includes information on the gestational age at birth [19], allowed determination of the day during gestation in which the prescription had been filled. Information on all hospital admissions are registered with the Danish National Patient Registry [20], along with International Classification of Diseases 10th revision (ICD-10) codes describing the underlying cause of the hospitalization.

The Danish educational registers are historically well preserved with near-complete information on the level of educational attainment for all Danish-born individuals from 1960 [21]. Furthermore, all examination grade marks at the final year of compulsory schooling (9th grade, children aged 15–16 years) have been recorded from the school year 2001/2002. In addition to educational data, we obtained intelligence test results from the national evaluation for military service (i.e., military conscription), which have been stored at the Danish Ministry of Defense since 2006. Military conscription is mandatory for Danish adolescent males the year they turn 18 years, unless exempt for a specified medical reason. The military intelligence test is highly correlated with a standard Wechsler Adult Intelligence Scale test [22] allowing estimation of IQ [23].

### Full-sibling cohort

Using information from the CRS, we selected all children born in Denmark from January 1, 1996 to December 31, 2003. Of these, we created a cohort of children who had one or more full-siblings born in the same period, and who were alive and had not emigrated by their 16th birthday. To investigate intelligence as assessed at evaluation for national military service, we selected boys from the cohort who had one or more full-brothers born in the same period, creating a nested full-brother sub-cohort. Cohort selection is further described in Fig A in S1 Appendix.

### Exposure

Using information on date of filled antimicrobial prescriptions and gestational age as an indicator of maternal infection during pregnancy, we categorized bi-weekly exposure periods during pregnancy (0–1 week of gestation, 2–3 week of gestation, etc.), in addition to a continuous exposure variable defined by the day of exposure during gestation. We defined exposure to systemic antimicrobials (e.g., sulfonamides) by ATC codes and a tablet formulation (see Table A in S1 Appendix for definitions of antimicrobial subtypes). Lastly, to assess infections requiring maternal hospitalization, we analogously conducted an analysis of maternal hospitalizations due to infections during pregnancy. Maternal hospitalization due to infection was defined by the date of in-patient hospital admission with a corresponding ICD-10 code for an infectious disease, as defined previously [24] (with calculous cholecystitis and uncomplicated appendicitis excluded), and only included if lasting more than one day. A diagnosis of infection in Danish hospital registries has previously been found to be highly predictive of an underlying infection [25]. For the few children with missing information on gestational age, mode imputation was used to estimate exposure period.

### Outcomes

We used examination grades from nationwide tests in written Danish (i.e., the principal school language) and written mathematics at the final year of compulsory schooling (9th grade, children aged 15–16 years) as outcomes. Both grades have been continuously given at yearly national examinations during the study period and the grades are ascertained by blinded assessors. The grades were standardized as z-scores according to year of examination. Additionally, in the nested full-brother sub-cohort we used intelligence test scores (standardized as z-score according to year of birth) given at the mandatory evaluation for national military service for boys, which correspond to IQ, as an outcome for boys in the full-brother cohort.

### Statistical analysis

As a basic model, adjusted mean difference in z-score according to exposure, for each of the three cognitive outcomes, was estimated using multivariable linear regression with adjustment for parental age at childbirth, parental educational achievement at the time of childbirth, and number of older siblings (covariates categorized as in Table 1). As a separate elaborated model, we further adjusted for shared family factors by conditioning on sibling membership, whereby unmeasured genetic and social factors shared among siblings were taken into account [26]. In this design, only sibling groups including both siblings with and without the exposure contribute to the estimation. Conditioning on sibling membership was achieved by subtracting sibling-specific means from the outcome and covariates before running the multivariable linear regression.

**Table 1:** Full-sibling cohort demographic information. Description of the 274,166 children in the full-sibling cohort, by sex, gestational age at birth, parental age at childbirth, parental educational level, number of older full-siblings, maternal filled antimicrobial prescription during pregnancy, and maternal in-patient hospitalization due to infection during pregnancy.

Characteristic	Full-sibling cohort^†^ (%)	
Sex		
Female	133,285	(48.6%)
Male	140,881	(51.4%)
Gestational age at birth (weeks)		
<37	19,161	(7.0%)
37–41	227,750	(83.1%)
≥42	20,254	(7.4%)
Missing information	7,001	(2.6%)
Number of older full-siblings		
0	112,503	(41.0%)
1	120,170	(43.8%)
2	31,514	(11.5%)
3	7,026	(2.6%)
≥4	2,953	(1.1%)
Maternal age at childbirth (years)		
<20	3,073	(1.1%)
20–24	35,920	(13.1%)
25–29	106,452	(38.8%)
30–34	95,360	(34.8%)
35–39	30,188	(11.0%)
≥40	3,173	(1.2%)
Maternal educational level^*^		
Primary education	55,520	(20.3%)
Upper secondary education	29,785	(10.9%)
Vocational education and training	93,827	(34.2%)
Short-term higher education	11,861	(4.3%)
Vocational bachelor education	55,078	(20.1%)
Academic bachelor’s degree	4,915	(1.8%)
Academic master’s degree	19,585	(7.1%)
PhD or other doctoral degree	796	(0.3%)
No maternal educational level registered	2,799	(1.0%)
Maternal filled antimicrobial prescription during pregnancy		
Any	80,817	(29.5%)
None	193,349	(70.5%)
Maternal in-patient hospitalization due to infection during pregnancy		
Any	5,628	(2.1%)
None	268,538	(97.9%)
Paternal age at childbirth (years)		
<20	701	(0.3%)
20–24	15,701	(5.7%)
25–29	76,183	(27.8%)
30–34	106,167	(38.7%)
35–39	54,152	(19.8%)
≥ 40	21,262	(7.8%)
Paternal educational level^*^		
Primary education	55,723	(20.3%)
Upper secondary education	21,391	(7.8%)
Vocational education and training	113,110	(41.3%)
Short-term higher education	17,856	(6.5%)
Vocational bachelor education	30,078	(11.0%)
Academic bachelor’s degree	4,364	(1.6%)
Academic master’s degree	25,895	(9.4%)
PhD or other doctoral degree	1,844	(0.7%)
No paternal educational level registered	3,905	(1.4%)

† The total birth cohort from which the full sibling cohort was sourced consisted of 529,465 children born from January 1, 1996 to December 31, 2003. For further description of cohort selection see Supplementary Figure 1 in S1 Text.

* Parental educational level defined as the highest educational level attained at the time of childbirth. Description and examples of specific educational categories are given in Supplementary Table 4. Missing parental educational level was imputed to primary education in analyses.

Exposure to antimicrobials and hospitalizations due to infection during pregnancy was modeled in multiple ways. Any antimicrobial or hospitalization exposure (binary), number of antimicrobial prescriptions or hospitalizations (categorical), and any filled prescription of a specific antimicrobial (binary), were all evaluated in separate analyses including one exposure at a time. Timing of antimicrobial prescriptions and hospitalizations was modeled by two separate approaches. In the first approach, we used a simultaneous inclusion of 21 continuous variables each representing a cohort member’s number of antimicrobial prescriptions or hospitalization exposures in bi-weekly periods (i.e., gestational weeks 0–1, …, ≥40). In this model, each antimicrobial prescription or hospitalization had an additive timing-specific contribution, and all contributions were summarized in the 21 variables. In the second approach, we modeled the additive contribution of each antimicrobial prescription or hospitalization more parsimoniously by modeling the timing (in days) using a natural cubic spline, and summarizing all contributions in a number of variables. The number of knots was chosen prior to analysis based on the number of filled antimicrobial prescriptions or hospitalizations, with more knots chosen for analyses of more frequent exposures. Analyses with all antimicrobial prescriptions and specifically β-lactams as exposure used knots in the 0.25, 0.50, 0.75, and 0.90 quantiles. Analyses with trimethoprim and sulfonamide as exposure used knots in the 0.33 and 0.66 quantiles. All remaining analyses used knots in the 0.25, 0.50, and 0.75 quantiles. Ninety-five percent confidence bands for the splines were obtained by transforming the confidence set for the full parameter vector by the spline function.

In sensitivity analyses, we investigated not adjusting for shared family factors in the full-sibling cohort, the effect of restricting to term births for gestational age at birth, the effect of extending to the complete population cohort, the effect of adjusting for maternal smoking during pregnancy, and the relative risks of achieving z-scores below the 5th percentile according to different exposures, estimated by odds ratios using conditional logistic regression with sibling-group as conditioning strata. Furthermore, to evaluate an effect of non-participation in school exams due to poor cognitive abilities, we imputed the lowest 1% percentile z-score for those cohort members who did not attend school exams. In the conditional logistic regression, asymptotically valid point-wise 95% confidence bands for the spline curves were obtained using the asymptotic normality of the estimated parameters.

Analyses were performed with R version 4.3.1 [27] using the lm function (multivariable linear regression) from the stats package, the clogit function (conditional logistic regression) from the survival package [28,29], and the ns function (natural cubic splines) from the splines package.

The study had a prospective analysis plan used for a funding application for the study (see S1 Text). Apart from the use of R instead of SAS for statistical analyses, no substantial changes were made to the analyses proposed compared to the analyses conducted. In addition, the study is reported as per the Reporting of Studies Conducted using Observational Routinely-Collected Data (RECORD) guideline (see S1 RECORD Checklist).

### Ethics approval

The study was conducted using deidentified information from Danish national registers and study participants were not contacted. According to Danish law, ethical approval is not required for such research. The study’s use of register data was covered by an approval from the Danish Data Protection Agency for register-based studies conducted by Statens Serum Institut (approval No 2015-57-0102).

## Results

Our full-sibling cohort included 274,166 children of which 48.6% were female and 41.0% had no older full-siblings (Table 1). The cohort was composed of 128,727 sibling groups. Of these children, 80,817 (29.5%) had a mother who filled a prescription for a systemic antimicrobial during their pregnancy, while 5,628 (2.1%) had a mother who was in-patient hospitalized due to an infection during their pregnancy (see Tables B and C in S1 Appendix for number of prescriptions and most frequent diagnoses). The most common maternal age at childbirth was 25–29 years (38.8%) and the most common maternal education level achieved at the time of childbirth was ‘vocational education and training’ (34.2%), which are educational programs giving professional qualifications without necessitating secondary education for entry, such as nursing aids (see Table D in S1 Appendix for more information on educational categories). Information on gestational age was available for 97.4% of the cohort, and 7.2% of these children were born prior to 37 + 0 gestational weeks.

**Fig 1:**
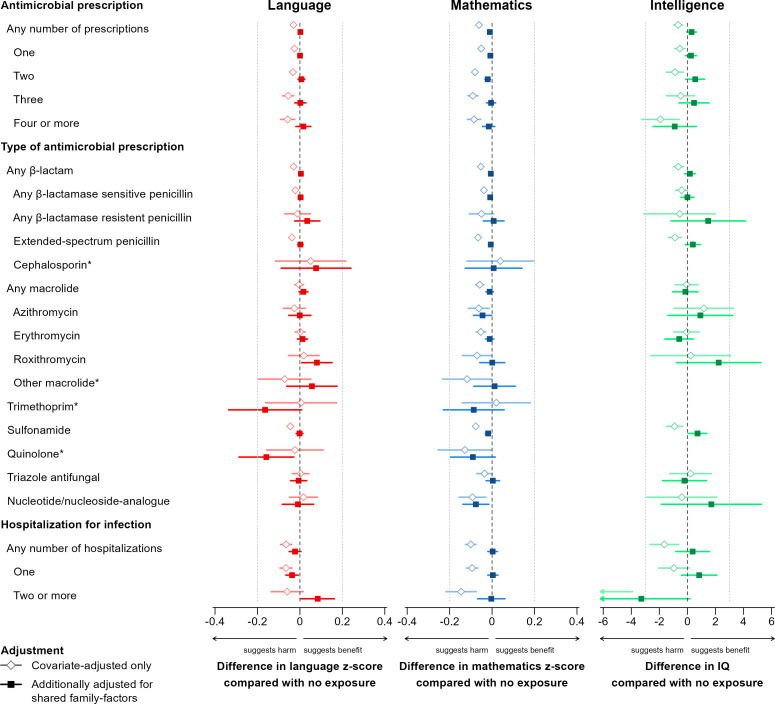
Difference in standardized grade (z-score) in language and mathematics for the full-sibling cohort and difference in IQ for the nested full-brother sub-cohort, by maternal exposure to infection during pregnancy compared with no exposure. All analyses are adjusted for maternal and paternal age at childbirth, maternal and paternal educational level, and number of older siblings. Estimates marked by diamonds (◊) are not adjusted for shared family factors (only covariate-adjusted), while estimates marked by squares (■) are additionally adjusted for shared family factors. Whiskers denote 95% confidence intervals. The estimates are additionally presented in tabular form in S1 Data. Asterisk (*) denote antimicrobial subtypes with 100 or fewer children exposed in the full-brother cohort, yielding uninformative IQ point estimates.

Investigating maternal infection during pregnancy, measured by filled antimicrobial prescriptions and in-patient hospitalization due to infection during pregnancy, we found no consistent lower school grades or IQ by number of antimicrobial prescriptions, type of antimicrobial prescription, or number of maternal hospitalizations for infection during pregnancy (Fig 1). Yet, we found a small, but statistically significant, lower grade specifically for mathematics associated with any filled antimicrobial prescription, one antimicrobial prescription, and two antimicrobial prescriptions (z-score differences of −0.01 (95% CI [−0.02, −0.00]; *p* = 0.003), −0.01, 95% CI [−0.02, −0.00]; *p* = 0.042, and −0.02 (95% CI [−0.03, −0.01]; *p* = 0.002), respectively), compared with no such exposure. However, the confidence intervals for these differences offer no statistical support for reduction of more than 2%, 2%, and 3% of a standard deviation in mathematics grade by exposure to any antimicrobial, one antimicrobial, and two antimicrobials, respectively, indicating minimal effects. For none of the 15 antimicrobial subtypes considered, associations with lower grades were found for both language and mathematics. Thus, while quinolone exposure was associated with lower language grades (z-score difference −0.16 (95% CI [−0.29, −0.03]; *p* = 0.017), it was not associated with lower mathematics grades (z-score difference −0.09 (95% CI [−0.20,0.02]; *p* = 0.094). Likewise, while exposure to azithromycin, sulfonamide, and nucleotide/nucleoside analogue were associated with small differences in mathematics grade (z-score differences of −0.05 (95% CI [−0.09, −0.00]; *p* = 0.040), −0.02 (95% CI [−0.03, −0.01]; *p* = 0.005), and −0.08 (95% CI [−0.14, −0.02]; *p* = 0.014)), they were not associated with lower language grades (z-score difference −0.00 (95% CI [−0.05,0.05]; *p* = 0.981), −0.00 (95% CI [−0.02,0.01]; *p* = 0.825), and −0.01 (95% CI [−0.08,0.07]; *p* = 0.804)). In the nested sub-cohort of full-brothers, we found no significant negative associations between antimicrobial prescriptions and maternal hospitalization for infection during pregnancy and IQ, but it should be noted that the confidence intervals for the rarer exposures are wide. In comparison, when not using a sibling design and adjusting only for measured covariates, exposure to both antimicrobials and maternal hospitalization during pregnancy was associated with substantial and consistent lower scores for all three cognitive outcomes (see estimates not adjusted for shared family factors in Fig 1).

**Fig 2:**
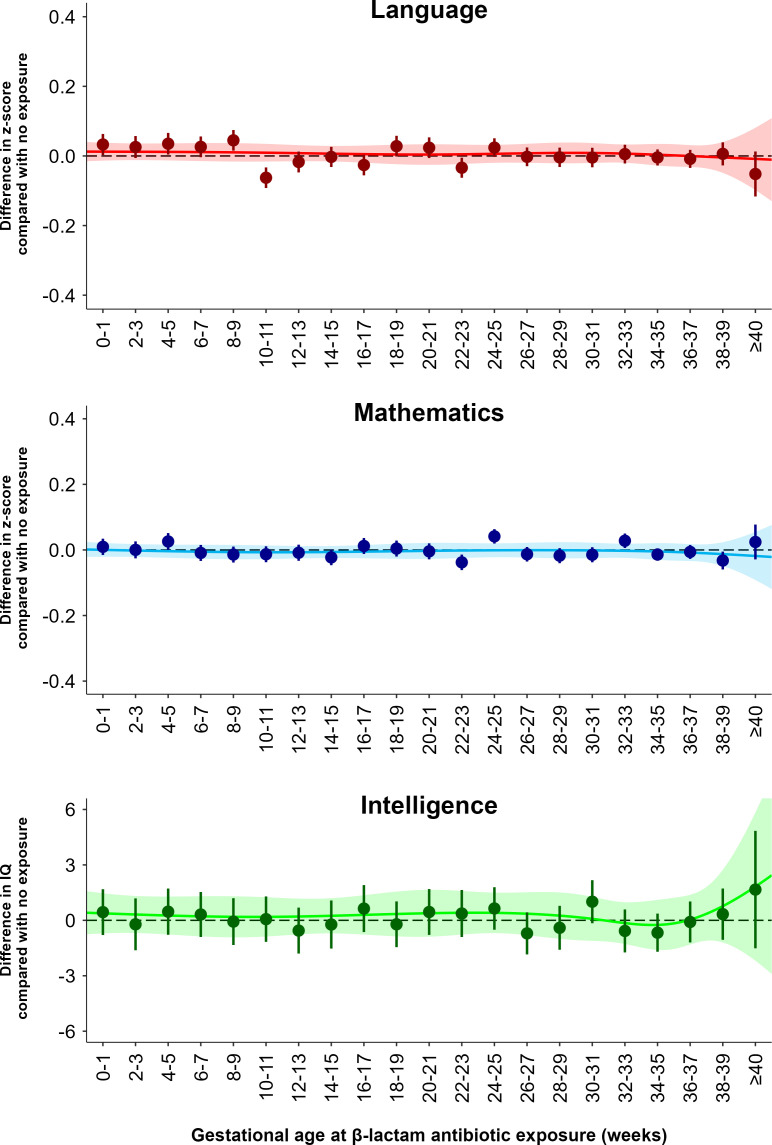
Difference in standardized grade (z-score) in language and mathematics for the full-sibling cohort and difference in IQ for the nested full-brother sub-cohort, given by gestational age at **β-lactam exposure compared with no exposure.** Analyses are adjusted for maternal and paternal age at childbirth, maternal and paternal educational level, number of older siblings, and shared family factors. Whiskers denote 95% confidence intervals. Lines denote natural cubic splines (modeled on the day of exposure) and the shaded areas denote the corresponding 95% confidence bands of these splines.

Examining exposure during different gestational weeks of fetal life to β-lactam antibiotics, the most common antimicrobial used during pregnancy, we found no lower level in language, mathematics, or IQ, when adjusted for covariates and shared family factors (Fig 2). This was found both when investigating exposure during bi-weekly gestational periods and when fitting a continuous spline based on day-specific exposure information during gestation. Likewise, we found no indications of period-specific effects of exposure to other common antimicrobials, such as macrolides, sulfonamides, and triazole antifungals (Fig B in S1 Appendix). We additionally conducted sensitivity analyses evaluating the role of gestational age at birth (Fig C in S1 Appendix) and maternal smoking during pregnancy (Fig D in S1 Appendix). Restricting our analyses of exposure to β-lactam antibiotics to children born within the 37th to the 41th gestational week, we found similar findings compared to the main analysis, suggesting no mediation of results through antimicrobial-exposed children being born pre-term or post-term. Using recorded information on maternal smoking during pregnancy (which were complete for 82% of women) for adjustment, we found indistinguishable results compared to the main analysis.

**Fig 3:**
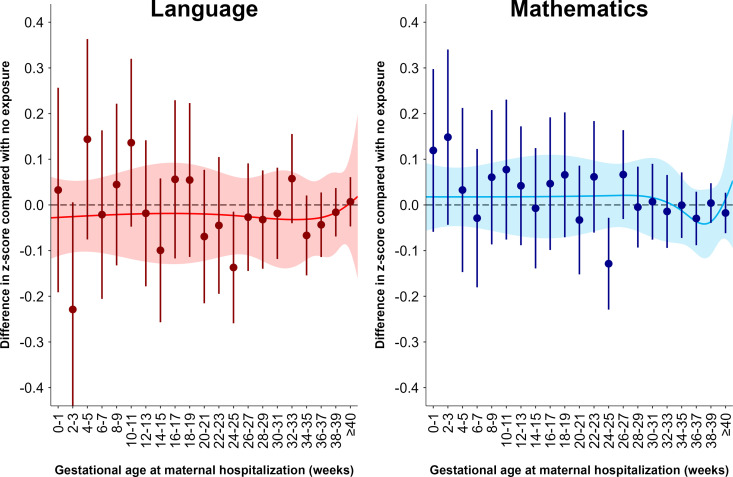
Difference in language and mathematics z-scores by gestational age at maternal in-patient hospitalization due to infection compared with no such exposure. Analyses are adjusted for maternal and paternal age at childbirth, maternal and paternal educational level, number of older siblings, and shared family factors. Whiskers denote 95% confidence intervals. Lines denote natural cubic splines (modeled on the day of exposure) and the shaded areas denote the corresponding 95% confidence bands of these splines.

To explore more severe infections during different gestational periods, we additionally investigated maternal in-patient hospitalization due to infections, as in-patient hospitalization indicates a more severe infection and most likely a more fulminant systemic inflammation. Nevertheless, this analysis did not indicate that severe infections during different periods of gestation are associated with lower school grades in language or mathematics, with the exception of significant negative association for maternal hospitalization in gestational weeks 24–25 (Fig 3). An analogous analysis in the full-brother cohort did neither indicate an association with IQ, although statistical power was limited and could only rule out large differences in IQ (Fig E in S1 Appendix). Restricting to children born at term, we found in a post-hoc analysis that the negative association found between maternal hospitalization during gestational weeks 24–25 and school grades disappeared (Fig F in S1 Appendix).

To evaluate the robustness of our approach, we compared covariate-adjusted estimates of the full-sibling cohort to a population cohort that also included singletons and children with only half-siblings (Fig G in S1 Appendix). We found no indication of any relevant differences between estimates in our full-sibling cohort and the population cohort. In addition, we estimated the risk of achieving outcome scores below the 5th percentile and found no indication of an increased risk of achieving scores below the 5th percentile when exposed to β-lactams, macrolides, or hospitalizations for infection during different periods of gestation, compared with no exposure (Fig H in S1 Appendix). Additionally, we found no indication of negative cognitive effects when cohort members who did not attend school exams were imputed with the lowest 1% percentile z-scores (Fig I in S1 Appendix).

## Discussion

Using a cohort of over 250,000 full-siblings with individual-level data on maternal prescriptions during their prenatal life, we found that filled antimicrobial prescriptions during gestation was not associated with school grades in language and mathematics at the final year of compulsory schooling. Likewise, no negative association was found with intelligence among adolescent males tested at mandatory evaluation for national military service. Finally, we found no consistent association between maternal in-patient hospitalizations for infection during pregnancy, which predominantly represent severe infections, and offspring school grades or intelligence, supporting a minimal impact of common maternal infections during pregnancy on cognitive abilities of the offspring.

Our study had the benefit of a large full-sibling cohort nested within a nationwide cohort of all children born in Denmark from 1996 to 2003. The population-based nature of the cohort and the full-sibling comparison allowed us to investigate the association between common maternal infections during pregnancy and offspring cognitive outcome on the population level, while controlling for both measured confounding (e.g., maternal age at birth) and unmeasured confounding due to shared family factors (i.e., genetics and social factors shared within families). In addition, a major advantage of our study was the prospectively collected exposure information, whereby we could precisely define the time point of exposure during gestation, avoiding recall bias and permitting examination of short time windows within gestation. Moreover, our study used cognitive outcome measures that were graded by blinded examiners, eliminating potential ascertainment bias due to knowledge of the preceding exposure.

Our study has five main limitations. First, we did not have information on medications given during in-patient hospitalizations. However, only 5,628 in-patient hospitalizations due to infectious disease occurred during gestation among 274,166 children (2.1% of pregnancies), why it was a rare occurrence during gestation, compared with the 29.5% of pregnancies in which there were dispensed systemic antimicrobials at community pharmacies. Furthermore, our analysis of the infections requiring maternal hospitalization did not indicate any substantial effects on cognitive outcomes, despite the more severe exposure. Second, our study was limited regarding information on the underlying pathogenic microorganisms, as the causative microorganism is rarely explored in non-severe common infections (e.g., uncomplicated pneumonia) and since microorganism-specific ICD-10 codes are rarely used for in-patient hospitalizations. In addition, our findings on common maternal infections in a high-income Nordic country might not be generalizable to developing countries or countries with a different infectious milieu (e.g., malaria and other tropical infections). Third, we did not have information on maternal fever during infections. Nevertheless, we did not find any indication of more adverse cognitive outcomes with more adverse exposure to infection (e.g., ranging from narrow-spectrum antibiotics prescriptions to broad-spectrum antibiotics prescriptions to in-patient hospitalization for infection), which are more likely to have been accompanied with fever. Fourth, use of the sibling design could potentially decrease generalizability (i.e., due to not including children without siblings) and increase the impact of misclassification. The similar results for mild infections, identified by prescriptions, and severe infections, identified by physician-defined hospital diagnosis codes, however, suggests limited bias due to misclassification of exposure. Lastly, an implicit bias of our study design is that only individuals who were born are included, while pre-natal infections potentially could have impaired brain development and resulted in early spontaneous abortion or intrauterine death. Yet, given the high frequency of infections during pregnancy, a dose-response association would be likely to have remained among the children born after the exposure, which our effect of number of prescriptions does not indicate.

Previous studies examining the association between maternal inflammatory response during pregnancy and long-term cognitive outcomes of the offspring have mostly been relatively small in size [4,7,9,10]. Furthermore, previous studies investigated broad exposure periods during pregnancy, such as exposure to infection during the entire pregnancy period or during a whole trimester [13]. Given the short critical developmental windows within pregnancy [30,31], our well-powered study provide substantial evidence to suggest limited impact of maternal infections across the many development windows in pregnancy. Furthermore, use of a full-sibling design allowed us to take shared family factors, such as both maternal and paternal intelligence, into account, whereas the few previous studies which considered shared family factors only considered factors clustered by sharing the same mother [8,13]. Nevertheless, akin to Choi and colleagues who found no association between maternal antibiotic exposure during pregnancy and intellectual disability in the offspring when using a sibling cohort, in contrast to a highly significant association when using a propensity score matched cohort [13], we found no association between maternal antimicrobial prescription and cognitive outcomes with respect to both schools grades and intelligence test results. An interesting aspect of the study by Choi and colleagues was also to investigate exposure to antibiotics beyond birth, in early infancy. Intriguingly, they found a markedly increased risk of intellectual disorder and language disorders (and also autism spectrum disorder) specifically in a subgroup with exposure to antibiotics within the first two months of life, both in their propensity score-matched cohort and sibling cohort. These findings indicate that very early postnatal common infections could have lasting negative effects on cognitive outcomes, and needs further study.

Our findings have several implications. First, our study suggests that the most commonly used antimicrobials in pregnancy (i.e., β-lactam antibiotics) are safe throughout gestation with regards to the cognitive outcome of the offspring. Second, our findings more broadly support that common maternal infections during pregnancy, either mild or severe, have very limited impact on offspring cognitive outcomes. Third, our study implies that the microorganisms causing clinically recognized infections during pregnancy are not associated with reduced long-term cognitive outcome of the offspring. Nevertheless, this pattern of association could potentially shift with a changing microbial environment, including introduction of novel infectious agents, as was seen with the Zika virus [3]. However, our study underscores that there is little reason to suspect strong effects on cognitive outcomes by a common immune response to infection, even in critical embryological periods during early gestation or during periods of substantial brain growth during late gestation.

Following the findings of us and others, it is our recommendation that future studies on the influence of maternal infections during pregnancy on offspring cognitive outcomes would benefit from using a sibling design to control for familial confounding not removed by typical covariate adjustment or propensity score matching. In addition, future studies might gain an advantage from further detailed information on maternal infections with positive microbiological cultures or confirmatory PCR analysis to pinpoint specific effects of individual infectious agents, not possible in our current study.

Taken together, our study does not support major effects of exposure to common maternal infections during gestation on offspring cognitive outcomes. Furthermore, the large statistical power of the study allows us to suggest that there does not exist critical time windows for fetal brain development that are sensitive to the general inflammatory response during infection. Lastly, our study underscores the importance of using a valid study design when investigating associations between an exposure during pregnancy and subsequent outcomes in offspring, as highly significant findings in covariate-adjusted analysis, disappeared when applying a full-sibling design.

## Supporting information

S1 Appendix **Table A.** Anatomical Therapeutic Chemical (ATC) codes used for defining antimicrobial subtypes. **Table B.** Total number of antimicrobial prescriptions during pregnancy in the full-sibling cohort. **Table C.** Most frequent infectious disease diagnoses (≥100 cases) used for in-patient hospitalizations during pregnancy in the full-sibling cohort. **Table D.** Description of educational categories. Detailed description of cohort selection (**Figure A**). Flowchart of cohort selection with selection criteria and number of individuals excluded in each step. Exposure to macrolide, sulfonamide, and triazole antifungals during pregnancy (**Figure B**). Difference in standardized grade (z-score) in language and mathematics for the full-sibling cohort and difference in IQ for the nested full-brother sub-cohort, given by gestational age at exposure to macrolide, sulfonamide, or triazole antifungal, respectively, compared with no such exposure. Analyses are adjusted for maternal and paternal age at childbirth, maternal and paternal educational level, number of older siblings, and shared family-factors. β-lactam exposure among children born at term (**Figure C**). Difference in standardized grade (z-score) in language and mathematics for the full-sibling cohort and difference in IQ for the nested full-brother sub-cohort, given by gestational age at β-lactam exposure compared with no exposure, among children born at term. Analyses are adjusted for maternal and paternal age at childbirth, maternal and paternal educational level, number of older siblings, and shared family-factors. Effect of additional adjustment for maternal smoking during pregnancy (**Figure D**). Difference in standardized grade (z-score) in language and mathematics for the full-sibling cohort and difference in IQ for the nested full-brother sub-cohort, given by gestational age at β-lactam exposure compared with no exposure. Analyses are adjusted for maternal and paternal age at childbirth, maternal and paternal educational level, number of older siblings, shared family-factors, with and without adjustment for maternal smoking during pregnancy. For missing smoking status mode imputation was used. Association between maternal hospitalization and offspring IQ (**Figure E**). Difference in IQ for the nested full-brother sub-cohort by gestational age at maternal hospitalization due to infection compared with no such exposure. The analysis is adjusted for maternal and paternal age at childbirth, maternal and paternal educational level, number of older siblings, and shared family-factors. Association between maternal hospitalization and school grades in language and mathematics among children born at term (**Figure F**). Difference in standardized grade (z-score) in language and mathematics for the full-sibling cohort given by gestational age at maternal hospitalization due to infection compared with no such exposure. Analyses are adjusted for maternal and paternal age at childbirth, maternal and paternal educational level, number of older siblings, and shared family-factors. Comparison of population cohort and full-sibling cohort estimates (**Figure G**). Difference in standardized grade (z-score) in language (A) and mathematics (B) by gestational age at β-lactam exposure compared with no exposure, comparing covariate adjusted estimates in the population cohort (who were alive and had not emigrated by their 16th birthday) and the nested full-sibling cohort. Analyses are adjusted for maternal and paternal age at childbirth, maternal and paternal educational level, and number of older siblings. Odds ratio (OR) of achieving a score below the fifth percentile (**Figure H**). Odds ratio of achieving below the 5% lowest score in language and mathematics for the full-sibling cohort and in IQ for the nested full-brother sub-cohort, given by gestational age at β-lactam exposure, macrolide exposure, or maternal hospitalization due to infection compared with no such exposure. Analyses are adjusted for maternal and paternal age at childbirth, maternal and paternal educational level, number of older siblings, and shared family-factors. Association between β-lactam exposure and school grades with imputation of missing grades (**Figure I**). Difference in standardized grade (z-score) in language and mathematics for the full-sibling cohort given by gestational age at β-lactam exposure compared with no exposure, with imputation of missing school grades with lowest 1% percentile z-score. Analyses are adjusted for maternal and paternal age at childbirth, maternal and paternal educational level, number of older siblings, and shared family-factors.
(DOCX)

S1 Data Difference in standardized grade (z-score) in language and mathematics for the full-sibling cohort and difference in IQ for the nested full-brother sub-cohort, by maternal exposure to infection during pregnancy compared with no exposure. All analyses are adjusted for maternal and paternal age at childbirth, maternal and paternal educational level, and number of older siblings. The estimates are also presented graphically in Fig 1. Blank cells denote antimicrobial subtypes with 100 or fewer children exposed in the full-brother cohort, yielding uninformative IQ point estimates.
(XLSX)

S1 Text Funding proposal, including prospective analysis plan for the study described in funding application to the Health Foundation. (PDF)

S1 RECORD Checklist RECORD Checklist. (PDF)

## Abbreviations

ATC: Anatomical Therapeutic Chemical
CI: confidence interval
CRS: Civil Registration System
ICD-10: International Classification of Diseases 10th revision
OR: odds ratio
