# Supplementary material for: Maternal infections during pregnancy and offspring cognitive outcome: A nationwide full-sibling cohort study
Source: PLoS Med. 2025 Jun 24;22(6):e1004657. doi: 10.1371/journal.pmed.1004657 (PMC12221162; doi:10.1371/journal.pmed.1004657)

**S1 Appendix**

Husby et al. Maternal infections during pregnancy and offspring cognitive outcome

[**Table A.** Anatomical Therapeutic Chemical (ATC) codes used for defining antimicrobial subtypes. 2](#_Toc200543339)

[**Table B.** Total number of antimicrobial prescriptions during pregnancy in the full-sibling cohort. 3](#_Toc200543340)

[**Table C.** Most frequent infectious disease diagnoses (≥100 cases) used for in-patient hospitalizations during pregnancy in the full-sibling cohort. 4](#_Toc200543341)

[**Table D.** Description of educational categories. 5](#_Toc200543342)

[Detailed description of cohort selection (**Fig A**) 6](#_Toc200543343)

[Exposure to macrolide, sulfonamide, and triazole antifungals during pregnancy (**Fig B**) 7](#_Toc200543344)

[β-lactam exposure among children born at term (**Fig C**) 8](#_Toc200543345)

[Effect of additional adjustment for maternal smoking during pregnancy (**Fig D**) 9](#_Toc200543346)

[Association between maternal hospitalization and offspring IQ (**Fig E**) 10](#_Toc200543347)

[Association between maternal hospitalization and school grades in language and mathematics among children born at term (**Fig F**) 11](#_Toc200543348)

[Comparison of population cohort and full-sibling cohort estimates (**Fig G**) 12](#_Toc200543349)

[Odds ratio (OR) of achieving a score below the fifth percentile (**Fig H**) 13](#_Toc200543350)

[Association between β-lactam exposure and school grades with imputation of missing grades (**Fig I**) 14](#_Toc200543351)

| **Table A. Anatomical Therapeutic Chemical (ATC) codes used for defining antimicrobial subtypes.** | |
| --- | --- |
| **Antimicrobial subtype** | **ATC codes used** |
| Any β-lactam | J01C*, J01D* |
| β-lactamase sensitive penicillin | J01CE* |
| β-lactamase resistant penicillin | J01CF* |
| Extended-spectrum penicillin | J01CA* |
| Cephalosporin | J01D* |
| Any macrolide | J01FA* |
| Azithromycin | J01FA10 |
| Erythromycin | J01FA01 |
| Roxithromycin | J01FA06 |
| Other macrolides | J01FA02, J01FA09 |
| Trimethoprim | J01EA* |
| Sulfonamide | J01EB* |
| Quinolone | J01MA* |
| Triazole antifungal | J02AC* |
| Nucleotide/nucleoside-analogue | J05AB* |
| Any antimicrobial | All of the above |

*For these ATC codes all sub-categories are included.

| Table B. Total number of antimicrobial prescriptions during pregnancy in the full-sibling cohort. | |
| --- | --- |
| **Antimicrobial subtype** | **Number of prescriptions** |
| Any antimicrobial | 122,735 |
| Any β-lactam | 87,106 |
| β-lactamase sensitive penicillin | 46,143 |
| β-lactamase resistant penicillin | 1,228 |
| Extended-spectrum penicillin | 39,547 |
| Cephalosporin | 160 |
| Any macrolide | 11,179 |
| Azithromycin | 1,483 |
| Erythromycin | 8,554 |
| Roxithromycin | 772 |
| Other macrolides | 370 |
| Trimethoprim | 143 |
| Sulfonamide | 20,080 |
| Quinolone | 230 |
| Triazole antifungal | 2,755 |
| Nucleotide/nucleoside-analogue | 1,242 |

| Table C. Most frequent infectious disease diagnoses (≥100 cases) used for in-patient hospitalizations during pregnancy in the full-sibling cohort. | | | |
| --- | --- | --- | --- |
| **Diagnostic code** | **Diagnosis** | **Number of patients** | **% of total** |
| DO231 | Infections of bladder in pregnancy | 605 | 25.17 |
| DA099 | Gastroenteritis or colitis, unspecified | 242 | 10.07 |
| DO230 | Pyelonephritis in pregnancy | 147 | 6.11 |
| DN300 | Acute cystitis | 146 | 6.07 |
| DA084 | Viral intestinal infection, unspecified | 124 | 5.16 |
| DJ189 | Pneumonia, unspecified | 118 | 4.91 |

| **Table D. Description of educational categories.** | | |
| --- | --- | --- |
| **Educational category** | **Description** | **Examples** |
| Primary education (i.e., 9^th^ grade) | 9th grade minimum educational level |  |
| Upper secondary education | Secondary education qualifying for higher education | Technical, commercial, academic high school |
| Vocational education and training | Educations giving professional qualifications, typically without necessitating secondary education for entry | Carpenter, electrician, nursing aid |
| Short-term higher education | Typical one to two-year educational programs giving professional qualifications | IT-worker, financial analyst |
| Vocational bachelor education | Typical two to four-year educational programs giving professional qualifications | Nurse, schoolteacher |
| Academic bachelor’s degree | Three-year academic education | Bachelor in economics and business administration, bachelor in English |
| Academic master’s degree | Two to three year academic education necessitating an academic bachelor degree | Dentist, medical doctor, priest |
| PhD or other doctoral degree | Postgraduate scientific university degree | PhD degree in physics, doctoral degree in veterinary medicine |

Detailed description of cohort selection (Fig A)

Flowchart of cohort selection with selection criteria and number of individuals excluded in each step.


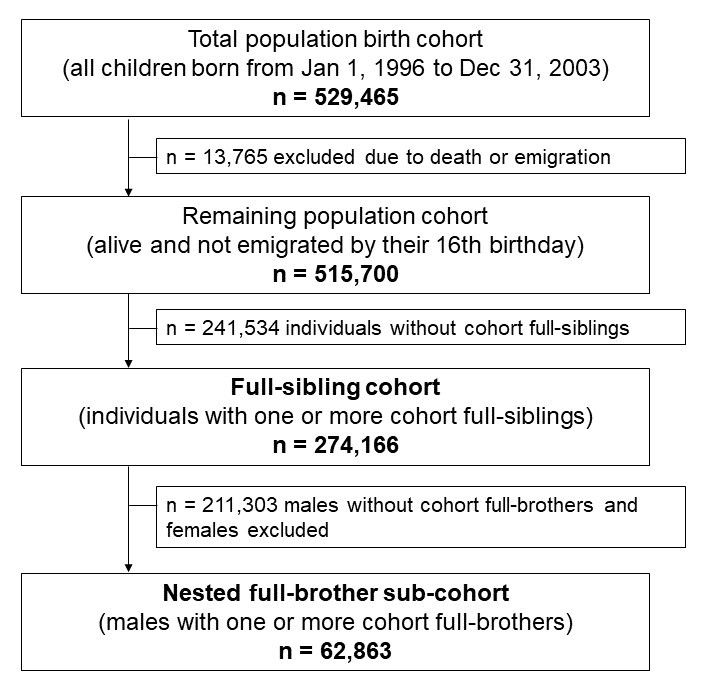


Exposure to macrolide, sulfonamide, and triazole antifungals during pregnancy (Fig B)

Difference in standardized grade (z-score) in language and mathematics for the full-sibling cohort and difference in IQ for the nested full-brother sub-cohort, given by gestational age at exposure to macrolide, sulfonamide, or triazole antifungal, respectively, compared with no such exposure. Analyses are adjusted for maternal and paternal age at childbirth, maternal and paternal educational level, number of older siblings, and shared family-factors.


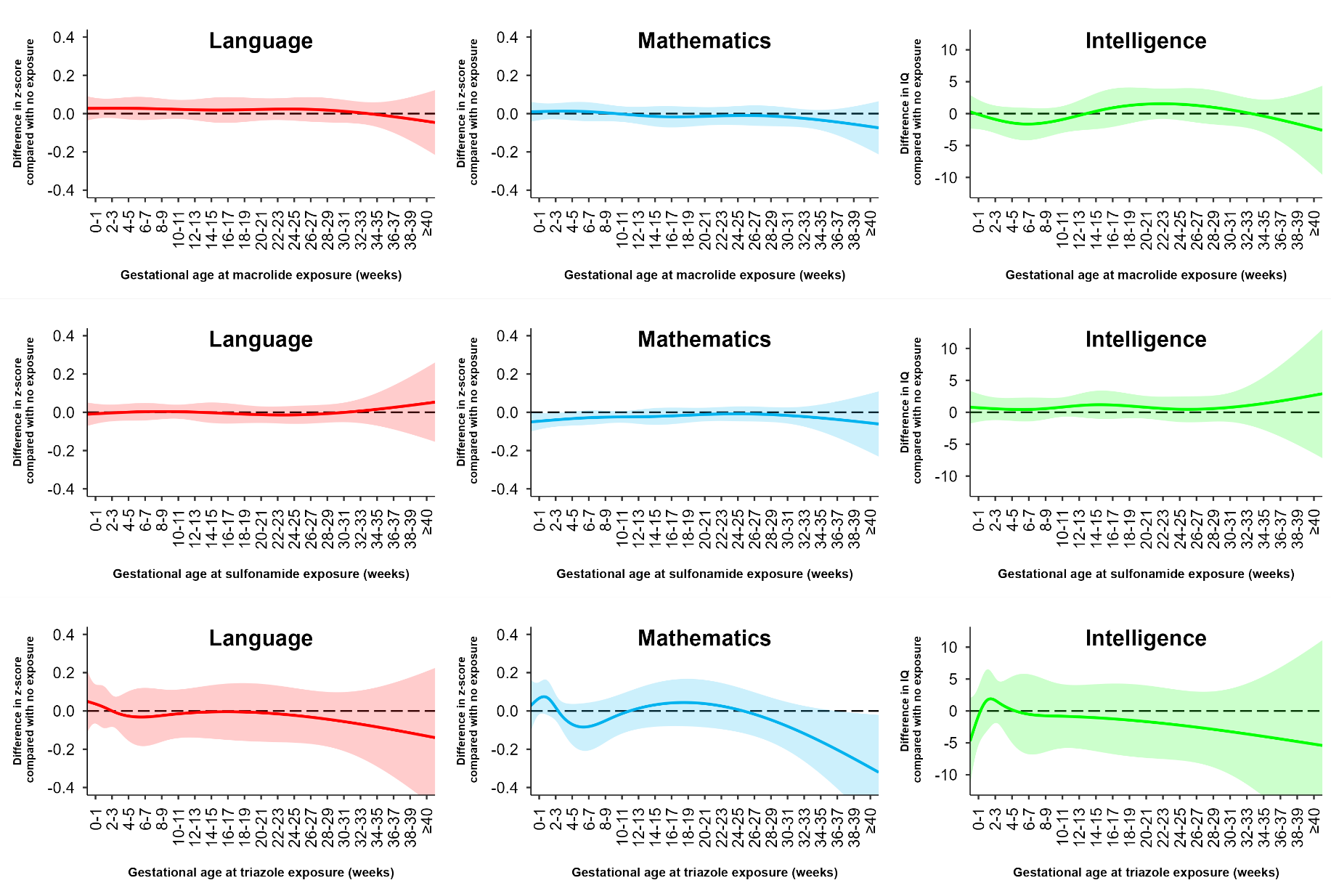


β-lactam exposure among children born at term (Fig C)

Difference in standardized grade (z-score) in language and mathematics for the full-sibling cohort and difference in IQ for the nested full-brother sub-cohort, given by gestational age at β-lactam exposure compared with no exposure, among children born at term. Analyses are adjusted for maternal and paternal age at childbirth, maternal and paternal educational level, number of older siblings, and shared family-factors.

**
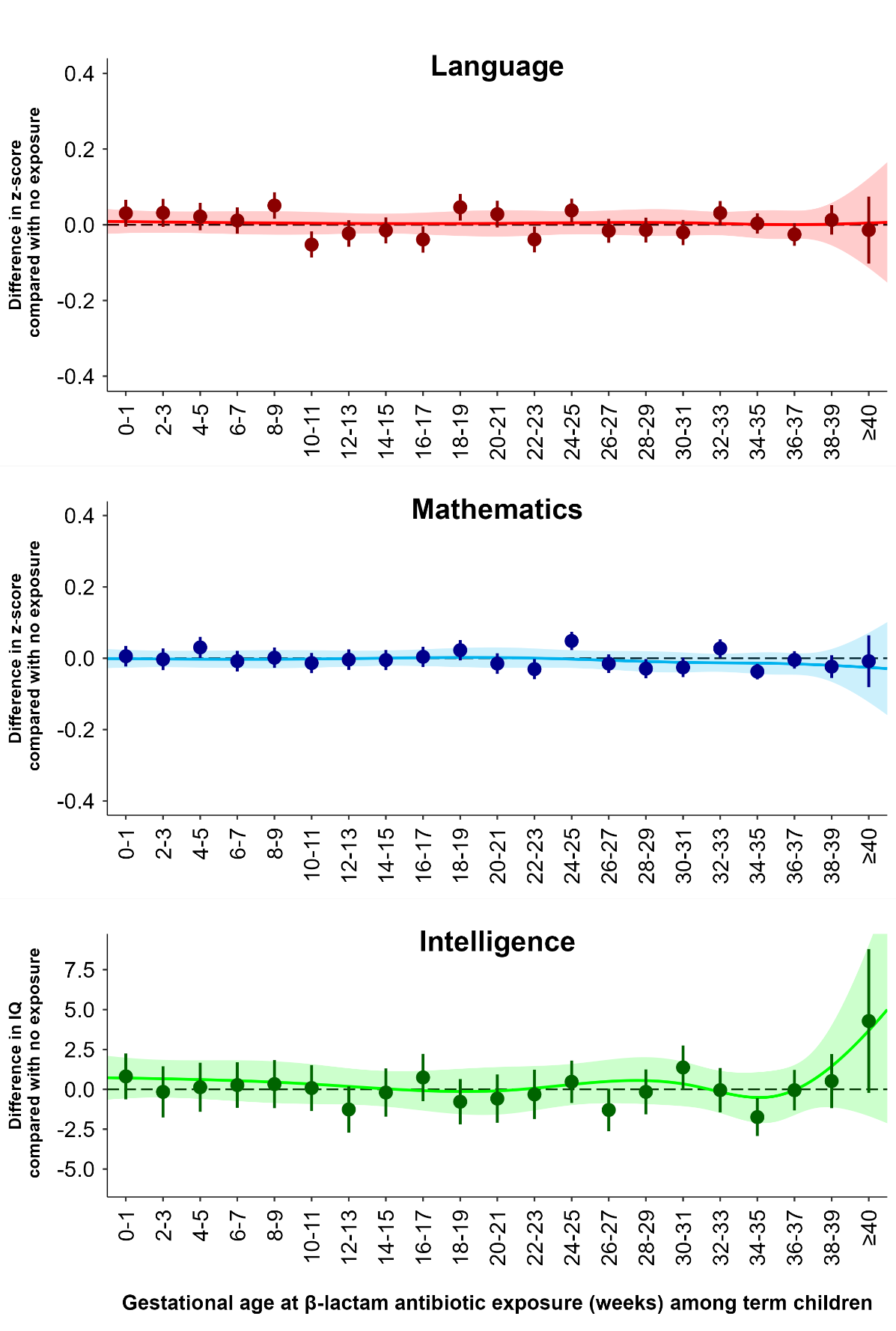
**

Effect of additional adjustment for maternal smoking during pregnancy (Fig D)

Difference in standardized grade (z-score) in language and mathematics for the full-sibling cohort and difference in IQ for the nested full-brother sub-cohort, given by gestational age at β-lactam exposure compared with no exposure. Analyses are adjusted for maternal and paternal age at childbirth, maternal and paternal educational level, number of older siblings, shared family-factors, with and without adjustment for maternal smoking during pregnancy. For missing smoking status mode imputation was used.

**
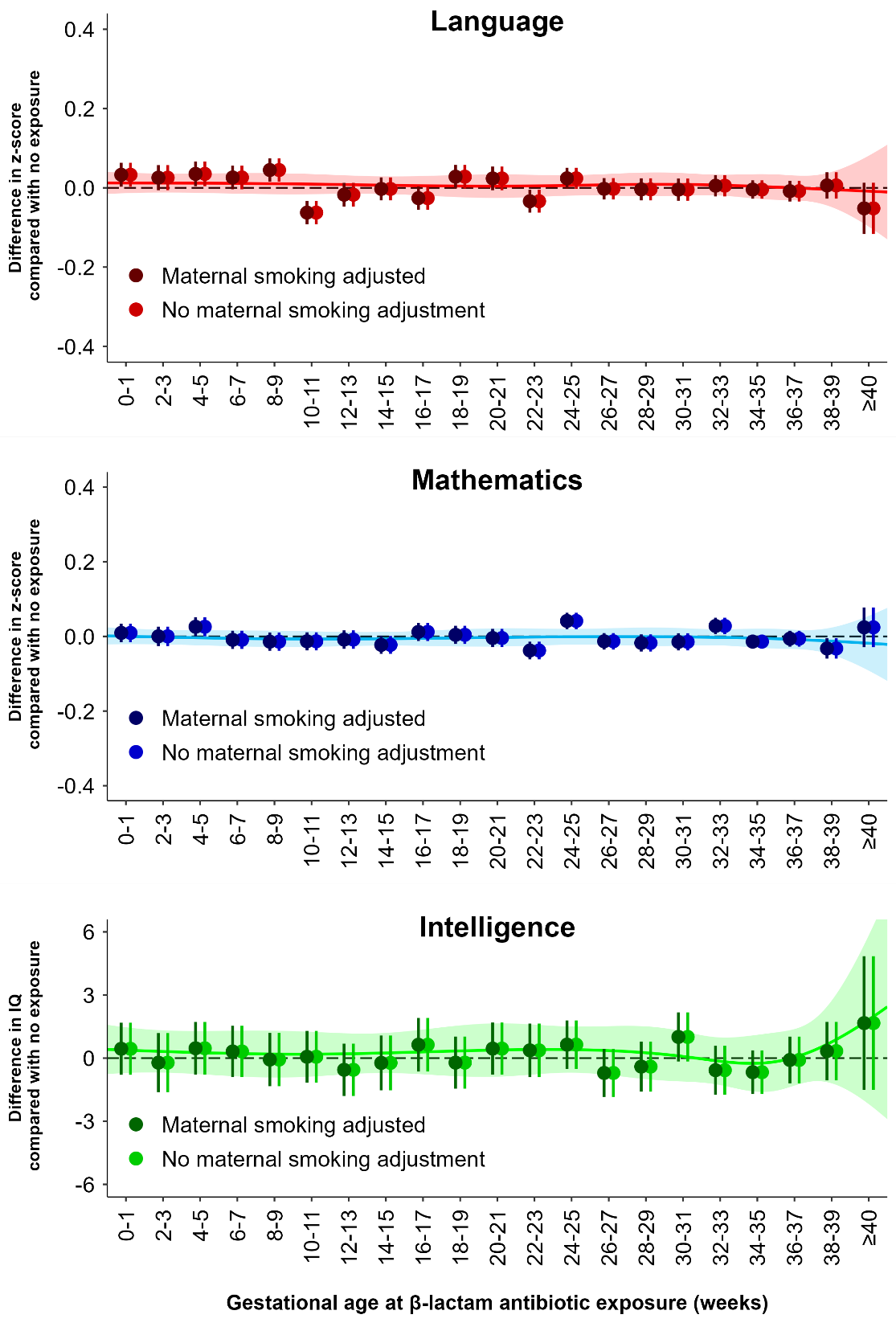
**

Association between maternal hospitalization and offspring IQ (Fig E)

Difference in IQ for the nested full-brother sub-cohort by gestational age at maternal hospitalization due to infection compared with no such exposure. The analysis is adjusted for maternal and paternal age at childbirth, maternal and paternal educational level, number of older siblings, and shared family-factors.

**
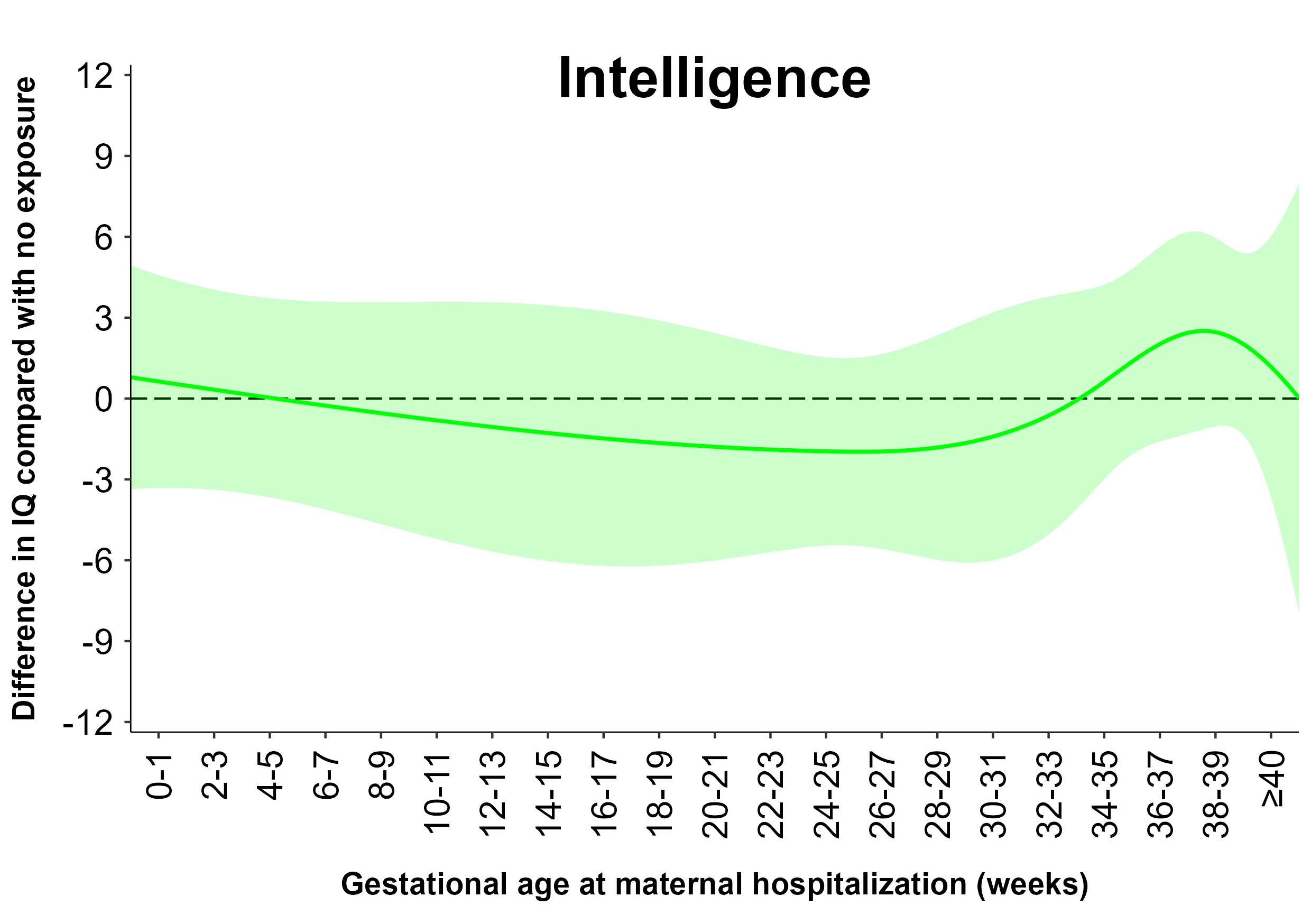
**

Association between maternal hospitalization and school grades in language and mathematics among children born at term (Fig F)

Difference in standardized grade (z-score) in language and mathematics for the full-sibling cohort given by gestational age at maternal hospitalization due to infection compared with no such exposure. Analyses are adjusted for maternal and paternal age at childbirth, maternal and paternal educational level, number of older siblings, and shared family-factors.

**
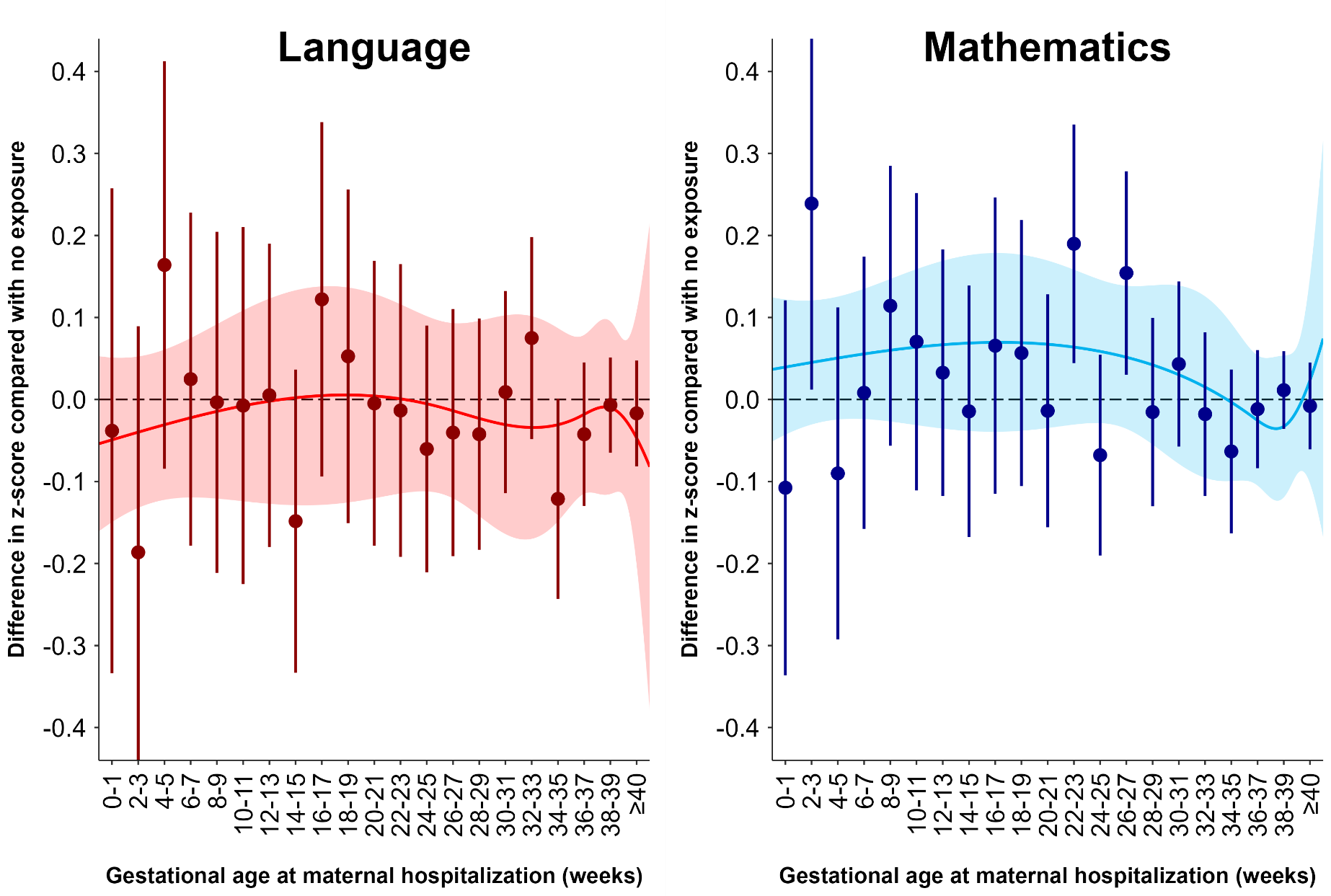
**

Comparison of population cohort and full-sibling cohort estimates (Fig G)

Difference in standardized grade (z-score) in language (A) and mathematics (B) by gestational age at β-lactam exposure compared with no exposure, comparing covariate adjusted estimates in the population cohort (who were alive and had not emigrated by their 16^th^ birthday) and the nested full-sibling cohort. Analyses are adjusted for maternal and paternal age at childbirth, maternal and paternal educational level, and number of older siblings.


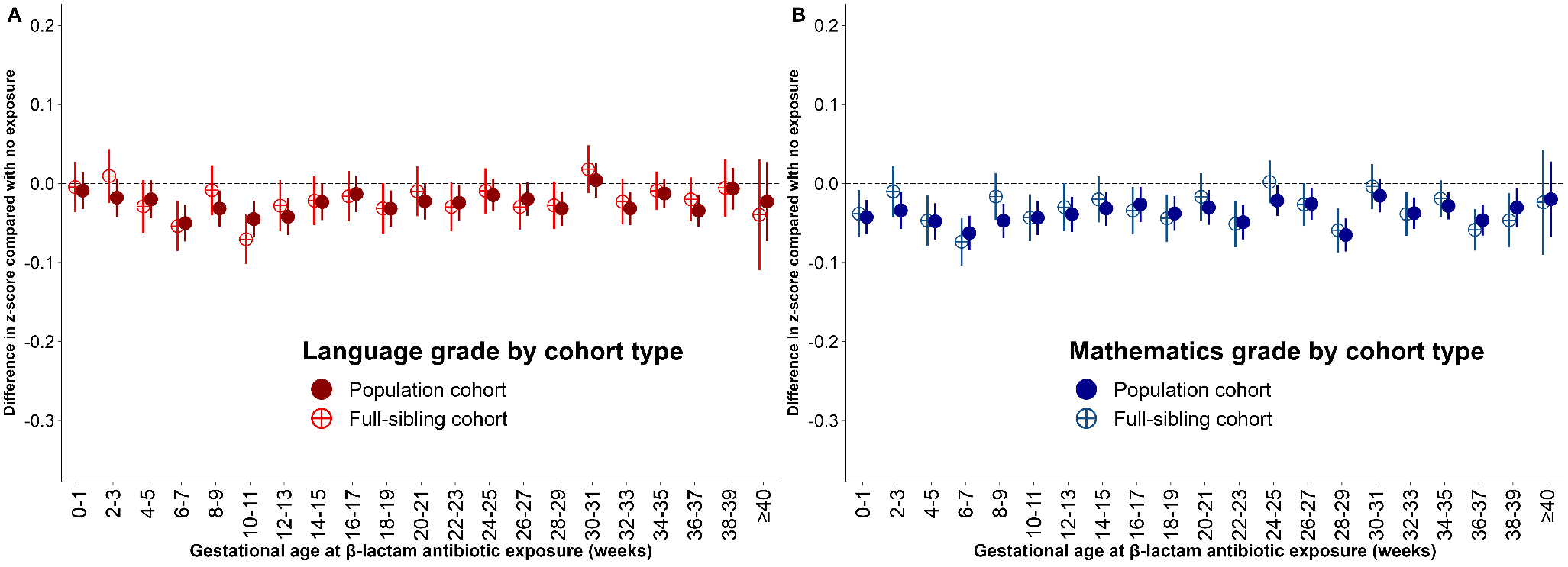


Odds ratio (OR) of achieving a score below the fifth percentile (Fig H)

Odds ratio (OR) of achieving below the 5% lowest score in language and mathematics for the full-sibling cohort and in IQ for the nested full-brother sub-cohort, given by gestational age at β-lactam exposure, macrolide exposure, or maternal hospitalization due to infection compared with no such exposure. Analyses are adjusted for maternal and paternal age at childbirth, maternal and paternal educational level, number of older siblings, and shared family-factors.


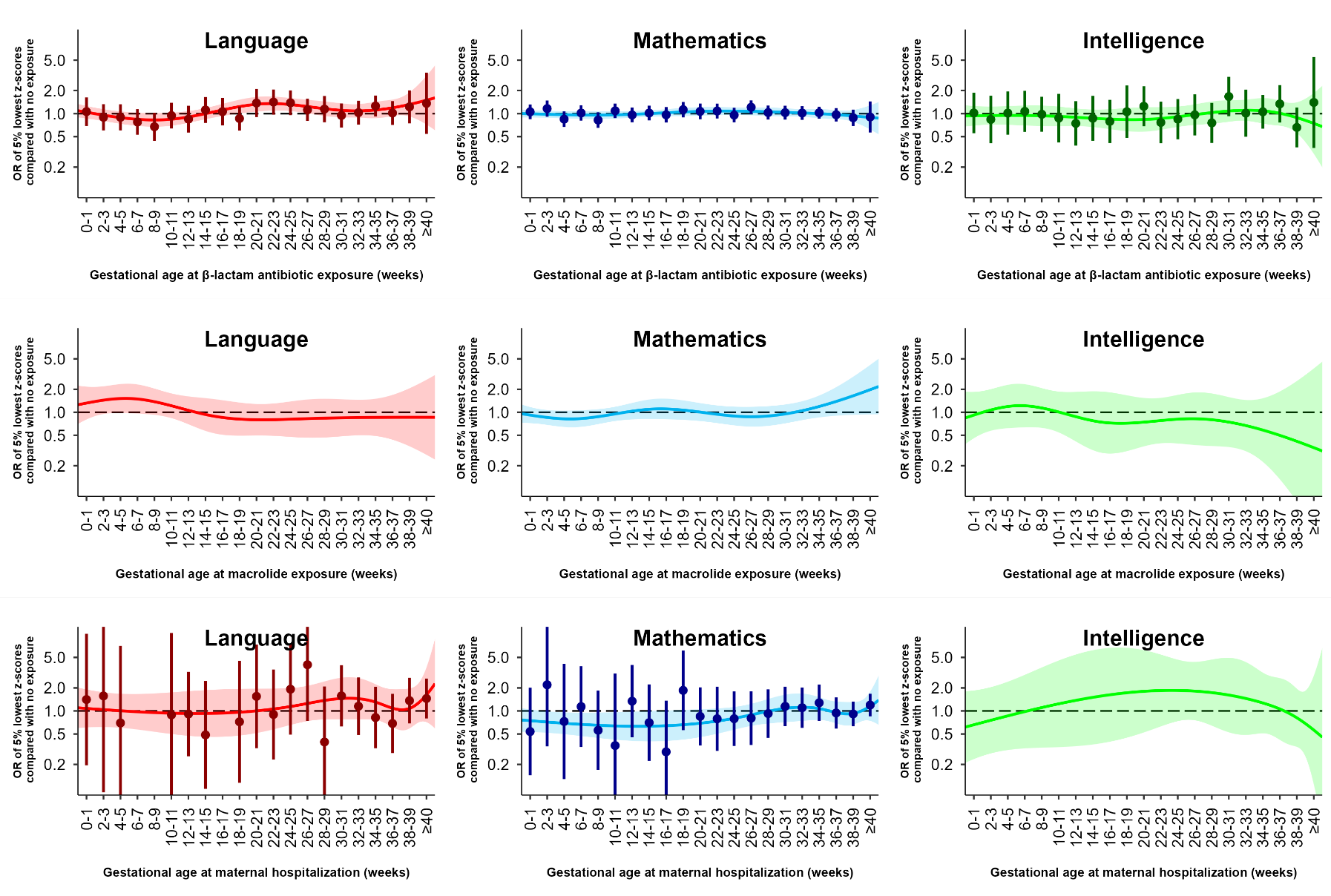


Association between β-lactam exposure and school grades with imputation of missing grades (Fig I)

Difference in standardized grade (z-score) in language and mathematics for the full-sibling cohort given by gestational age at β-lactam exposure compared with no exposure, with imputation of missing school grades with lowest 1% percentile z-score. Analyses are adjusted for maternal and paternal age at childbirth, maternal and paternal educational level, number of older siblings, and shared family-factors.


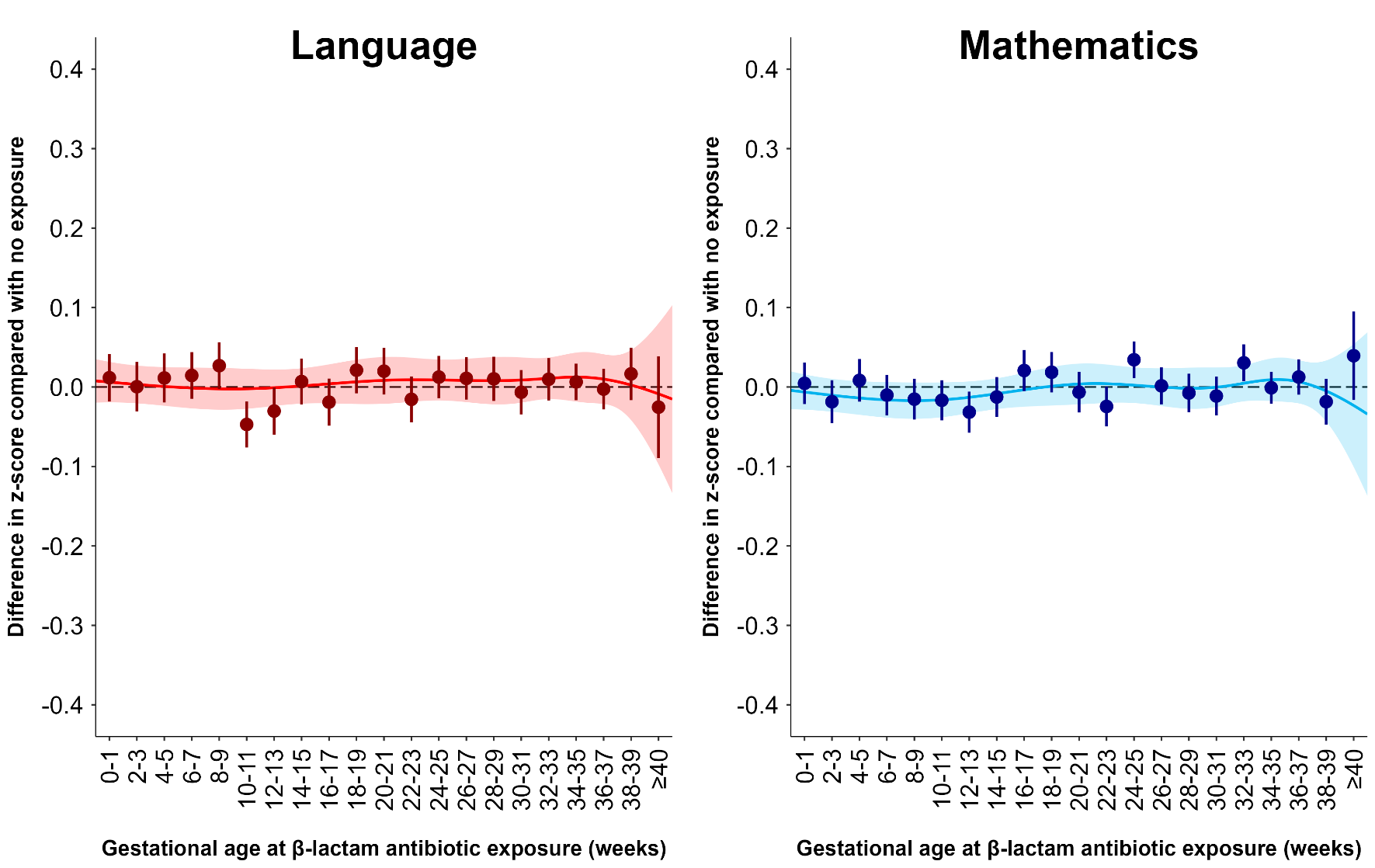

Supplement: S1 Appendix — Table A. Anatomical Therapeutic Chemical (ATC) codes used for defining antimicrobial subtypes. Table B. Total number of antimicrobial prescriptions during pregnancy in the full-sibling cohort. Table C. Most frequent infectious disease diagnoses (≥100 cases) used for in-patient hospitalizations during pregnancy in the full-sibling cohort. Table D. Description of educational categories. Detailed description of cohort selection (Figure A). Flowchart of cohort selection with selection criteria and number of individuals excluded in each step. Exposure to macrolide, sulfonamide, and triazole antifungals during pregnancy (Figure B). Difference in standardized grade (z-score) in language and mathematics for the full-sibling cohort and difference in IQ for the nested full-brother sub-cohort, given by gestational age at exposure to macrolide, sulfonamide, or triazole antifungal, respectively, compared with no such exposure. Analyses are adjusted for maternal and paternal age at childbirth, maternal and paternal educational level, number of older siblings, and shared family-factors. β-lactam exposure among children born at term (Figure C). Difference in standardized grade (z-score) in language and mathematics for the full-sibling cohort and difference in IQ for the nested full-brother sub-cohort, given by gestational age at β-lactam exposure compared with no exposure, among children born at term. Analyses are adjusted for maternal and paternal age at childbirth, maternal and paternal educational level, number of older siblings, and shared family-factors. Effect of additional adjustment for maternal smoking during pregnancy (Figure D). Difference in standardized grade (z-score) in language and mathematics for the full-sibling cohort and difference in IQ for the nested full-brother sub-cohort, given by gestational age at β-lactam exposure compared with no exposure. Analyses are adjusted for maternal and paternal age at childbirth, maternal and paternal educational level, number [file pmed.1004657.s001.docx]
