# Supplementary material for: Maternal infections during pregnancy and offspring cognitive outcome: A nationwide full-sibling cohort study
Source: PLoS Med. 2025 Jun 24;22(6):e1004657. doi: 10.1371/journal.pmed.1004657 (PMC12221162; doi:10.1371/journal.pmed.1004657)
Supplement: S1 Text — (PDF) [file pmed.1004657.s003.pdf]

Week-specific antibiotic use in pregnancy and offspring cognitive outcome: A Nationwide Sibling Cohort Study

It is well-known that certain rare infections during pregnancy can cause severe fetal brain damage and led to life-long mental impairment. However, it is unclear if common maternal infections during short critical periods of fetal brain development also restrict long-term cognitive development. Using gestational week-specific information on all filled antibiotic prescriptions during the fetal life of over 500,000 children, linked to information on school grades, we will investigate if common maternal infections and the accompanying inflammatory response impact long-term brain function of the offspring.

BACKGROUND

The fetal brain undergoes massive development during pregnancy, with neuronal synaptogenesis reaching peak intensity with the formation of 40,000 new synapses every second[1]. Multiple studies have suggested that common maternal infections during pregnancy and their inflammatory responses (e.g., fever) negatively impact fetal brain development and later cognitive abilities[2,3]. Given that cognitive abilities are closely linked to the risk of premature death[4] and lifelong quality-of-life[5], it is an important factor to investigate and identification of preventive measures could have large societal effects.

However, previous population-based cohort studies have found inconsistent results, with one cohort study finding no impact of fever during pregnancy on school performance[6], while a separate cohort study found hospital-contacts for any infection during pregnancy profoundly decreased offspring intelligence[7]. Nevertheless, neither of the previous large cohort studies had exact assessment of which time-period during pregnancy the infection occurred and none took family-based factors into account.

Using the Danish Civil Registration System and the Danish National Prescription Register, containing information on all prescribed pharmaceuticals in Denmark from 1995[8], we will create a nationwide cohort of more than 500,000 individuals, with week-specific information on maternal antibiotic prescriptions during pregnancy. As antibiotic prescriptions are filled at the precise time of infection, we will have exact knowledge of which gestational week of pregnancy that the mother had an infection, allowing highly detailed assessment of the exposure.

To assess long-term effects of infections on cognitive function in offspring, we will use data on national school marks given to all pupils in the final year of mandatory public schooling (da. '*Folkeskolens afgangsprøve*'), which since 2002 have been registered at Statistics Denmark[9]. For a

subset of the cohort, we will additionally take advantage of the compulsory intelligence test used at male conscription in Denmark[10] to investigate adolescent intelligence. Furthermore, linking to information on siblings in the Civil Registration System, will allow us to take account of underlying family-based factors that are associated with both propensity to infection and later life cognitive abilities, which often can bias analyses of such exposures.

MATERIALS

The Danish Civil Registration System (CRS) will form the backbone of the cohort, providing family linkage, in addition to linkage to other registers. Information on school grades and conscription intelligence test results is obtained from the Student Register at Statistics Denmark, and The Danish Ministry of Defense, respectively. Information on the pharmaceutical exposures and morbidities will be collected from The National Prescription Registry, The Danish National Patient Register, The Danish Cancer Registry, The National Health Service Registry, and the Medical Birth Register. Socioeconomic covariates, such as educational level, employment, and household disposable income will be sourced from Statistics Denmark.

METHODS

Full-sibling cohort

Using the register information described above, we will create a nationwide population cohort of all individuals born in Denmark from October 1, 1995 to December 31, 2003. We will restrict the cohort to all individuals who were alive and had not emigrated by their 15th birthday. In addition, to take account of within-family factors, we will narrow our cohort to individuals who had one or more full-siblings (i.e., the same mother and father), yielding a full-sibling study cohort. In a nested male sub-cohort, we will restrict the final full-sibling cohort to only males who had one or more full-brothers, within the cohort, who were alive and had not emigrated by their 18th birthday.

Exposure definition

Filled prescriptions from the Registry of Medicinal Products Statistics to mothers of the cohort members before, during, and after pregnancy will be used to define the exposure. We will primarily define the exposure as a filled prescription for a systemic antibacterial (ATC group J01), to ensure that the antibiotics was given for a systemic illness with an inflammatory response. In additional analyses, subtypes of antibacterials and other systemic antibiotics will be investigated (i.e., systemic antiviral and systemic antimycotics).

Outcome definition

For school outcome we will use a standardized grade average for grades in written language and mathematics, which are graded by blinded examiners, at examinations at the final year of mandatory schooling in Denmark[9]. For intelligence, we will use test results from Børge Priens intelligence test which has a high correlation with standard intelligence tests and has been used for over 50 years for conscription of adult men into the Danish armed forces[10]. Furthermore, the test is known to have high validity and re-test reliability[11].

Statistical methods

We will used fixed effect regression to take the sibling design into account and thereby indirectly adjust for unmeasured family factors. The association between weekly antibiotic exposure during fetal life (coded as exposed and non-exposed for each gestational week) and cognitive outcomes, will directly be adjusted for sex, relative birthweight, gestational age, maternal smoking, parental age at birth, parental educational level at childbirth, and number of older siblings. We will use a unique sibling identifier for each full-sibling group (i.e., all children registered with the same mother and father). Estimation will be performed using PROC GLM in SAS© version 9.4 using the ABSORB statement to take the sibling design into account. Sensitivity analysis by subtype of antibiotic and similar analysis for antiviral and antimycotic medications will be conducted. Furthermore, to disentangle the effects of a specific pathogen from the effects of a general inflammatory response (e.g. fever), we will also analyze effects of maternal hospital-contacts for specific infections coded with relevant ICD-10 codes.

Statistical power considerations

In preliminary analysis of statistical power, we have found that there are more 2500 pregnant women exposed to systemic antibiotics (by filled prescriptions) in each gestational week from conception to the 37th week of pregnancy in the described time period. Given that over 90% of the offspring attend school exams, this will provide a large study set for estimating weekly effects of fetal exposure to maternal infection.

Benefits of using a sibling design

The added value of using a sibling design in comparison with a regular cohort design is illustrated in Figure 1 shown below, which presents preliminary data from a study on gestational age at birth and subsequent cognitive outcomes.

As shown in Figure 1, inclusion of adjustment for sibling factors markedly attenuates negative effects of preterm and early term birth on school grades, in comparison with regular cohort adjustment for potential strong confounders (e.g., sex, maternal educational level, maternal smoking, and birthweight). It is therefore conceivable that other pregnancy exposures, such as exposure to antibiotics, also will be confounded by family-based factors.

FIGURE 1. Preliminary data on school grades in Danish (A) and Mathematics (B) by gestational age at birth relative to 40 gestational weeks, with respectively no adjustment (○), adjustment for covariates only (⊕), and adjustment for covariates and sibling factors (●).

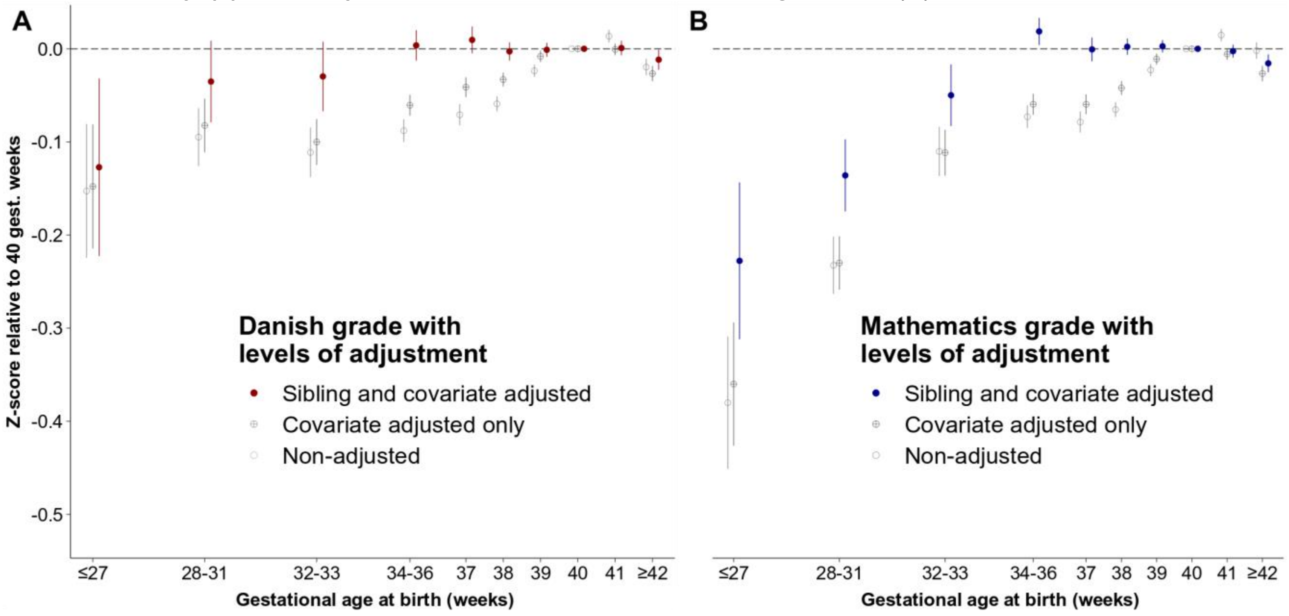

TIME FRAME AND COLLABORATION

The project is planned to take 12 months and will be conducted under the leadership of applicant Anders Husby, postdoc at the Department of Epidemiology Research at Statens Serum Institut (SSI). Starting June 1, 2022 to May 31, 2023, a junior epidemiologist/medical doctor/bioinformatician will carry out data classification, analysis, and drafting of the manuscript under the supervision of Anders Husby. Chief Statistician Jan Wohlfahrt (also SSI) and Professor Mads Melbye (Faculty of Medicine and Health Sciences, Norwegian University of Science and Technology) who have many years of high-quality register-research experience will additionally co-supervise the project.

DISSEMATION OF RESULTS

The results from the cohort will be disseminated to the general public, public health officials, and researchers by press-releases, social media postings (e.g., twitter-tutorials written for the general public and researchers), and peer-reviewed scientific articles.

BUDGET

We apply Helsefonden for 12 months of salary for a junior epidemiologist/medical doctor/bioinformatician, in addition to data storage costs at Statistics Denmark while running the project. The data required for the project is already available at our Statistics Denmark server why the project will be able to start right away.

REFERENCES

1. Tau GZ, Peterson BS. Normal development of brain circuits. *Neuropsychopharmacology*. 2010;35(1):147-168. doi:10.1038/npp.2009.115
2. Dreier JW, Andersen AMN, Berg-Beckhoff G. Systematic review and meta-analyses: Fever in pregnancy and health impacts in the offspring. *Pediatrics*. 2014;133(3):e674-e688. doi:10.1542/peds.2013-3205
3. Antoun S, Ellul P, Peyre H, et al. Fever during pregnancy as a risk factor for neurodevelopmental disorders: results from a systematic review and meta-analysis. *Mol Autism*. 2021;12(1):1-13. doi:10.1186/S13229-021-00464-4/FIGURES/10
4. Batty GD, Deary IJ, Gottfredson LS. Premorbid (early life) IQ and Later Mortality Risk: Systematic Review. *Ann Epidemiol*. 2007;17(4):278-288. doi:10.1016/j.annepidem.2006.07.010
5. Johnson W, Corley J, Starr JM, Deary IJ. Psychological and Physical Health at Age 70 in the Lothian Birth Cohort 1936: Links With Early Life IQ, SES, and Current Cognitive Function and Neighborhood Environment. *Heal Psychol*. 2011;30(1):1-11. doi:10.1037/a0021834
6. Dreier JW, Berg-Beckhoff G, Andersen PK, Andersen AMN. Prenatal Exposure to Fever and Infections and Academic Performance: A Multilevel Analysis. *Am J Epidemiol*. 2017;186(1):29-37. doi:10.1093/AJE/KWX053
7. Benros ME, Sørensen HJ, Nielsen PR, Nordentoft M, Mortensen PB, Petersen L. The Association between Infections and General Cognitive Ability in Young Men – A Nationwide Study. *PLoS One*. 2015;10(5):e0124005. doi:10.1371/JOURNAL.PONE.0124005
8. Pottegård A, Schmidt SAJ, Wallach-Kildemoes H, Sørensen HT, Hallas J, Schmidt M. Data Resource Profile: The Danish National Prescription Registry. *Int J Epidemiol*. 2016;25(3):dyw213. doi:10.1093/ije/dyw213
9. Schmidt LB, Corn G, Wohlfahrt J, Melbye M, Munch TN. School performance in children with infantile hydrocephalus: A nationwide cohort study. *Clin Epidemiol*. 2018;10:1721-1731. doi:10.2147/CLEP.S178757
10. Teasdale TW. The Danish Draft Boards intelligence test, Børge Priens Prøve: Psychometric properties and research applications through 50 years. *Scand J Psychol*. 2009;50(6):633-638. doi:10.1111/j.1467-9450.2009.00789.x
11. Teasdale TW, Hartmann PVW, Pedersen CH, Bertelsen M. The reliability and validity of the Danish Draft Board Cognitive Ability Test: Børge Prien's Prøve. *Scand J Psychol*. 2011;52(2):126-130. doi:10.1111/j.1467-9450.2010.00862.x
